# Lymph node detection of monoclonal T‐cell population correlates with poorer prognosis in mycosis fungoides and Sèzary syndrome

**DOI:** 10.1111/bjh.70051

**Published:** 2025-08-11

**Authors:** Denis Miyashiro, Hebert Fabricio Culler, Jade Cury‐Martins, Bruno C. Souza, Luís Alberto de Pádua Covas Lage, Juliana Pereira, José A. Sanches

**Affiliations:** ^1^ Department of Dermatology University of São Paulo Medical School São Paulo Brazil; ^2^ Discipline of Hematology, Hemotherapy and Cell Therapy, Department of Internal Medicine University of São Paulo Medical School São Paulo Brazil

**Keywords:** clonality, cutaneous lymphomas, mycosis fungoides, prognostic factors, Sèzary syndrome

To the Editor,

Mycosis fungoides (MF) is the most frequent cutaneous T‐cell lymphoma, and Sézary syndrome (SS) is its leukaemic variant.^1^ Most MF cases have an indolent course, with a 5‐year overall survival (OS) between 88.0% and 94.6% for early stage (IA–IIA). However, advanced stage (IIB–IVB) has poorer prognoses, with a 5‐year OS between 41.0% and 68.5%.^2^ Efforts have been made to identify characteristics that predict the progression of the disease and the risk of death.^3^ Extracutaneous involvement is associated with a worse prognosis. Lymph nodes are the most frequently extracutaneous involved site.^4^ Sometimes, lymph node tumour burden is scarce, and histopathology may not detect an aberrant cell. Searching for T‐cell clones may detect monoclonal neoplastic cells even if there are low amounts of malignant cells.^5^ We aimed to evaluate the role of the current lymph node staging system and T‐cell clonality status and correlate them with prognosis.

All patients with MF/SS followed at the University of São Paulo Medical School, Brazil, between 1987 and 2023, were analysed, but only patients with lymph node biopsies, whose histopathology and clonality status were available, were included. The current tumor‐node‐metastasis‐blood (TNMB) staging system for lymph nodes (N) was used. N0: no clinically abnormal peripheral lymph nodes; N1: dermatopathic lymphadenopathy or occasional atypical lymphocytes isolated or aggregated into three to six cell clusters; N2: aggregates of atypical lymphocytes without alteration of lymph node architecture; N3: partial/complete effacement of lymph node architecture. If a monoclonal population was detected, ‘b’ was added to the N stage; if no monoclonal population was detected, ‘a’ was added.^6^ To support this classification, immunohistochemistry was employed.

All patients were primarily treated with agents available in the Brazilian Public Health System, including topical therapies (e.g. corticosteroids) and phototherapy (UVA or UVB) for early stage, and biological agents (α‐interferon, acitretin), monochemotherapy (low‐dose methotrexate, chlorambucil, gemcitabine) and CHOP‐like chemotherapy for advanced‐stage MF/SS. No patient was upfront treated with new agents, including bexarotene (topical or oral), histone deacetylase inhibitors, brentuximab vedotin, mogalulizumab or photopheresis.

For T‐cell clonality assay, DNA extraction using QIAamp DNA mini kit (Qiagen, Germany) was conducted in 10 cuts of formalin‐fixed paraffin‐embedded (FFPE) tissue containing 5 μm thickness. The cuts were placed in 1.5‐mL microtubes followed by the addition of 200 μL of tissue lysis buffer and 20 μL of proteinase K. This solution was incubated at 56°C at 500 rpm for 16 h in ThermoMixer® (Eppendorf, Germany) and centrifuged at 6000× *g* for 1 min to remove droplets from the inside of the microtube. After this step, 200 μL of AL buffer was added, followed by homogenization for 15 s in a vortex and incubation at 70°C for 10 min in ThermoMixer®. After adding 200 μL of absolute ethanol, the protocol was performed according to the manufacturer's recommendation.

For each multiplex polymerase chain reaction (PCR), 100 ng of previously extracted and quantified DNA, 1.25 μL of 10× buffer, 0.4 μL of 50 mM MgCl_2_, 0.25 μL of 10 mM DNTP, 0.5 μL of a mix of the seven oligonucleotides (IDT, Iowa, USA) at 10 pmol/μL, 0.2 μL (1 U) of Platinum Taq DNA Polymerase (Invitrogen, California, USA) and enough ultrapure water for a final volume of 12.5 μL were used. PCR amplification was carried out using a Veriti thermocycler (Applied Biosystems, Foster City, CA) under the following conditions: initial denaturation at 94°C for 3 min; 40 cycles of denaturation at 95°C for 1 min, annealing at 61.8°C for 30 sec, and extension at 72°C for 30 sec; followed by a final extension at 72°C for 10 min and a hold at 4°C. The oligonucleotide sequences were designed according to Shadrach and Warshawsky.^7^ For sequencing, the 1:10 PCR product was diluted, and 1 μL was added to a mixture containing 8.5 μL of formamide (Applied Biosystem, California, USA) and 0.5 μL of GeneScan Rox (Applied Biosystems, California, USA), totalling 10 μL. The mixtures were placed in a 96‐well plate specific for the ABI 3500 Genetic Analyser sequencer (Applied Biosystems, California, USA) and subjected to the following injection conditions: 8‐s injection, 19.5 kV pre‐run voltage, 1.6 kV injection voltage, dye set D, POP‐7 polymer and 60°C oven temperature. Data were analysed, and monoclonality was determined by visual examination of electropherograms using GeneMapper software v4.1 (Applied Biosystem, California, USA).^7^

Statistical analyses were performed with STATA version 13 (STATA Corp., Texas, USA). Qualitative data are shown as frequencies and percentages and were analysed with chi‐squared or Fisher's exact tests. Survival curves were plotted according to the Kaplan–Meier method and analysed with the log‐rank test, and univariate and multivariate analyses were performed with the Cox proportional hazards model. Statistical significance was considered when *p* ≤ 0.05.

Among 745 MF/SS patients, 563 (75.6%) were staged as N0, 132 (17.7%) N1, 13 (1.7%) N2 and 37 (5.0%) N3. T‐cell clonality in lymph nodes was assessed in 48 patients, particularly after 2009, when this assay was routinely incorporated in our centre (Figure 1A, Table 1). Of these, 14 (29.2%) were N1a; 21 (43.7%) N1b; 0 N2a; 4 (8.3%) N2b; 0 N3a; and 9 (18.7%) N3b. Considering MF patients, 11 (39.3%) were stage N1a; 10 (35.7%) N1b; 1 (3.6%) N2b; and 6 (21.4%) N3b. Considering SS patients, 3 (15.0%) were stage N1a; 11 (55.0%) stage N1b; 3 (15.0%) stage N2b; and 3 (15.0%) stage N3b. There was no statistically significant difference between N stage and MF or SS diagnosis (*p* = 0.144).

**FIGURE 1:**
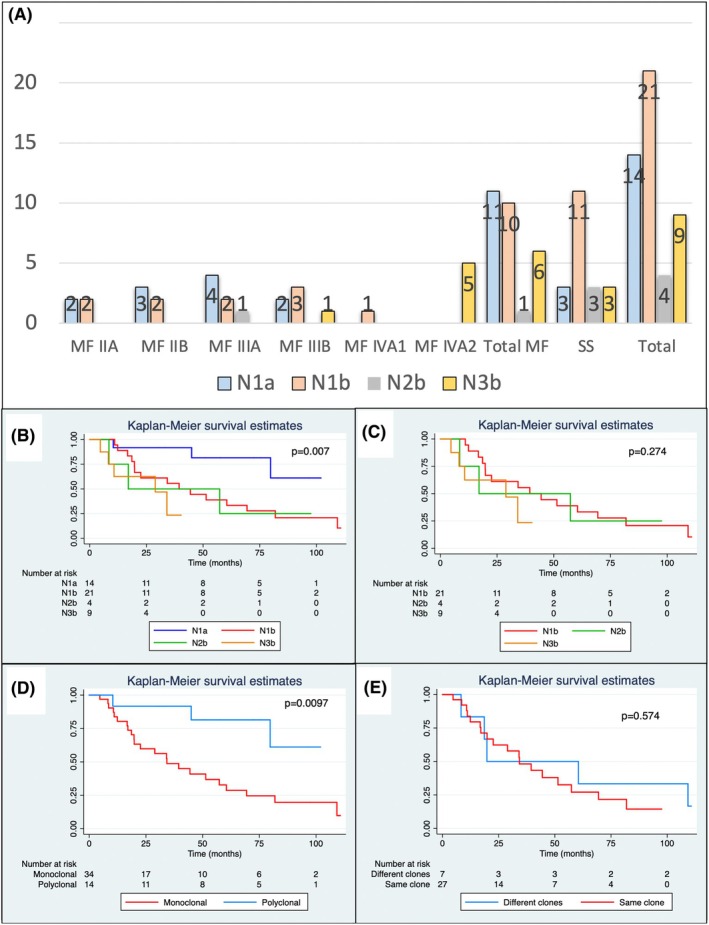
N stage according to diagnosis and MF staging (A). Survival curves considering N stage and T‐cell clonality on the lymph nodes considering all patients (B). When excluding N1a from the analysis, there was no statistically significant difference between survival curves (C). Considering only the T‐cell clonality status, a significant difference was observed (D). Among the patients with a monoclonal population detected on the lymph nodes, the presence of the same or different clones in other sites (blood or skin) did not show different survival curves (E).

**TABLE 1:** N stage according to diagnosis and MF staging.

	MF							SS	Total
	IIA	IIB	IIIA	IIIB	IVA1	IVA2	Total		
N1a (%)	2 (50.0)	3 (60.0)	4 (57.1)	2 (33.3)	0	0	11 (39.3)	3 (15.0)	14 (29.2)
N1b (%)	2 (50.0)	2 (40.0)	2 (28.6)	3 (50.0)	1 (100)^a^	0	10 (35.7)	11 (55.0)	21 (43.7)
N2a (%)	0	0	0	0	0	0	0	0	0
N2b (%)	0	0	1 (14.3)	0	0	0	1 (3.6)	3 (15.0)	4 (8.3)
N3a (%)	0	0	0	0	0	0	0	0	0
N3b (%)	0	0	0	1 (16.7)	0	5 (100)	6 (21.4)	3 (15.0)	9 (18.7)
Total	4	5	7	6	1	5	28	20	48

Abbreviations: MF, mycosis fungoides; SS, sézary syndrome.

^a^ MF patient with multiple infiltrated plaques covering >10% of body surface area (T2b) and B2.

For the whole MF/SS cohort, 5‐year OS was 94.2% for N0; 61.7% for N1; 38.6% for N2; and 25.3% for N3 (*p* < 0.0001). However, when considering the T‐cell clonality status, 5‐year OS was 81.8% for N1a; 33.3% for N1b; 25.0% for N2b; and 23.4% for N3b (*p* < 0.0001) (Figure 1B). When excluding N1a from the analysis, the 5‐year OS among N1b, N2b and N3b did not differ significantly (*p* = 0.274) (Figure 1C). Considering only the clonality status, patients with lymph node monoclonal T‐cell population had 5‐year OS of 28.7%, and patients with no monoclonal population had 5‐year OS of 81.8% (*p* = 0.0097) (Figure 1D). The same monoclonal T‐cell population was detected in the lymph nodes and blood and/or skin in 27 (79.4%) patients, and the clones were different in 7 (20.6%). However, there were no differences in survival curves between patients with the same or different clones (*p* = 0.574) (Figure 1E).

On multivariate analysis, the detection of monoclonal T‐cell population on lymph nodes had a higher risk of death (hazard ratio 3.99, 95% confidence interval 1.06–14.91), emerging as an independent prognostic marker (*p* = 0.040), even considering the current staging system (‘N’ staging and TNMB staging) and the diagnoses of MF or SS (Table 2).

**TABLE 2:** Univariate and multivariate analyses.

	Hazard ratio	95% confidence interval		*p*‐value
Univariate analysis				
Female vs. male^a^	0.60^a^	0.44	0.82	0.001
Age	1.04	1.03	1.05	<0.001
MF vs. SS^b^	5.70	3.91	8.30	<0.001
Staging	1.57	1.48	1.68	<0.001
Lymph node clonality	4.33	1.29	14.51	0.018
Lymph node staging	2.36	2.06	2.70	<0.001
Multivariate analysis				
Male vs. female	1.22	0.52	2.90	0.648
Age	1.02	0.99	1.05	0.160
MF vs. SS	0.74	0.24	2.29	0.601
Staging	1.04	0.70	1.55	0.851
Lymph node clonality	3.99	1.06	14.91	0.040
Lymph node staging	1.09	0.50	2.40	0.823

Abbreviations: HR, hazard ratio; MF, mycosis fungoides; SS, sézary syndrome.

^a^ Female had lower HR compared to male.

^b^ SS had higher HR compared to MF.

There are few studies evaluating the role of T‐cell clonality in the lymph nodes of MF/SS patients.

Kubica et al. evaluated 176 SS patients. In 13 (18.3%), there was no lymph node involvement; in 37 (52.1%), only lymph node histology revealed involvement by SS; in three patients (4.2%), only molecular lymph node data were consistent with SS; and in 18 patients (25.4%), histology and molecular data were consistent with SS.^8^ Similarly, in our SS patients, three (15.0%) had no lymph node involvement (N1a), and in six (30.0%), both histology and clonality were consistent with lymphomatous involvement (N2b or N3b). However, Kubica et al. did not observe an association between a positive clone in lymph node and increased risk of death (hazard ratio 1.85, confidence interval 0.93–3.68, *p*‐value 0.08).^8^ Different from our study, these authors used both Southern blot and PCR in different timelines, but we used only PCR, which is more sensitive to identify molecular abnormalities. This different approach makes the comparison between both studies more difficult.^9^

Fraser‐Andrews et al. evaluated 60 MF/SS patients with lymph node biopsies. A monoclonal population was detected in 6/19 (31.6%) of MF patients with uninvolved or limited histological lymph node involvement and in 13/14 (92.8%) of MF patients with advanced histological involvement.^10^ A similar result was obtained by Assaf et al., who detected a monoclonal population in 7/14 (50%) of non‐involved lymph nodes and in 22/22 (100%) of involved lymph nodes.^5^ We also observed that, among 21 MF patients with no lymph node histological involvement (N1), 10 (47.6%) had monoclonal populations, and among seven patients with N2 or N3, all had monoclonal populations (100%).

Considering SS patients, we detected a monoclonal T‐cell population in 17/20 (85.0%) lymph node biopsies, similar to the literature (81.5%).^10^

In previous studies, monoclonal population in the lymph nodes was associated with poorer prognosis, with a median survival of 54 months from diagnosis, compared to patients with nonclonal populations, with a median survival of 284 months. However, multivariate analysis did not reveal a significant association between lymph node clonality and survival.^10^ In another study considering only histologically non‐involved lymph nodes, detection of monoclonal population was also associated with poorer prognosis (median probability of survival of 74 months) compared to patients with nonclonal populations (all seven patients were alive).^5^ These findings are similar to our observations. Nonetheless, this is the first study to demonstrate an association between lymph node monoclonality and poorer prognosis after multivariate analysis.

The N1 stage includes dermatopathic lymphadenopathy and occasional atypical lymphocytes, isolated or aggregated into three to six cell clusters.^6^ We did not review each individual lymph node biopsy, and we cannot determine if monoclonal T cells were observed more frequently in the N1 stage containing small clusters of atypical lymphocytes compared to dermatopathic lymphadenopathy. A revision of the current staging system could address the relevance of clonality and whether N1 with dermatopathic lymphadenopathy differs in prognosis from those with atypical lymphocytes, either isolated or in small clusters.

Limitations of the study include its retrospective design, single‐centre cohort and lack of external validation, which may affect the generalizability of our findings. In addition, the small number of N2 and N3 cases may limit the statistical power.

In this large cohort, lymph node staging was essential to define prognosis. The incorporation of clonality contributed to the prediction of prognosis, especially in patients with N1. The current clinical staging system (IA–IVB) does not consider lymph node clonality status. However, according to our findings, N1a had a better prognosis, and N1b prognosis did not differ significantly from N2b and N3b. We suggest that more studies are needed to elucidate if T‐cell clonality should be incorporated in the staging classification and if T‐cell clonality in lymph node core biopsy in N0 stage patients could provide prognostic information.

## AUTHOR CONTRIBUTIONS

D.M. designed the research, analysed the data, performed the study and wrote the manuscript; H.F.C. and L.A.P.C.L. provided and analysed the clonality data; J.C. and B.C.S. assisted in research and data analysis; J.P. provided and analysed the clonality data and supervised the research; J.A.S. designed the research, analysed the data and supervised the research.

## FUNDING INFORMATION

No funding was received for the study.

## CONFLICT OF INTEREST STATEMENT

The authors declare no competing financial interests.

## ETHICS STATEMENT

All necessary approvals were obtained from our ethics committee, and the study was undertaken in accordance with the Declaration of Helsinki.

## PATIENT CONSENT STATEMENT

All patients provided written informed consent for personal data analysis for research purposes.

## Data Availability

For original data, please contact Denis Miyashiro, denisrmiyashiro@gmail.com.
